# Backward Locomotor Treadmill Training Differentially Improves Walking Performance across Stroke Walking Impairment Levels

**DOI:** 10.3390/brainsci12020133

**Published:** 2022-01-19

**Authors:** Oluwole O. Awosika, Dorothy Chan, Heidi J. Sucharew, Pierce Boyne, Amit Bhattacharya, Kari Dunning, Brett M. Kissela

**Affiliations:** 1 Department of Neurology and Rehabilitation Medicine, University of Cincinnati, Cincinnati, OH 45221, USA; chandh@mail.uc.edu (D.C.); KISSELBM@UCMAIL.UC.EDU (B.M.K.); 2 Cincinnati Children’s Hospital Medical Center, Division of Biostatistics and Epidemiology, Cincinnati, OH 45229, USA; Heidi.Sucharew@cchmc.org; 3 Department of Pediatrics, College of Medicine, University of Cincinnati, Cincinnati, OH 45267, USA; 4 Department of Rehabilitation, Exercise and Nutrition Sciences, University of Cincinnati, Cincinnati, OH 45221, USA; boynepe@UCMAIL.UC.EDU (P.B.); DUNNINKK@UCMAIL.UC.EDU (K.D.); 5 EDDI Lab—Early Detection of Degenerative Disorders & Innovative Solutions, Department of Environmental Health, College of Medicine, University of Cincinnati, Cincinnati, OH 45267, USA; BHATTAAT@UCMAIL.UC.EDU

**Keywords:** backward locomotion, post-stroke walking rehabilitation, gait rehabilitation, backward treadmill training, walking impairment, stroke walking severity

## Abstract

Background: Post-stroke walking impairment is a significant cause of chronic disability worldwide and often leads to loss of life roles for survivors and their caregivers. Walking impairment is traditionally classified into mild (>0.8 m/s), moderate (0.41–0.8 m/s), and severe (≤0.4 m/s), and those categorized as “severe” are more likely to be homebound and at greater risk of falls, fractures, and rehospitalization. In addition, there are minimal effective walking rehabilitation strategies currently available for this subgroup. Backward locomotor treadmill training (BLTT) is a novel and promising training approach that has been demonstrated to be safe and feasible across all levels of impairment; however, its benefits across baseline walking impairment levels (severe (≤0.4 m/s) vs. mild–moderate (>0.4 m/s)) have not been examined. Methods: Thirty-nine adults (>6 months post-stroke) underwent 6 days of BLTT (3×/week) over 2 weeks. Baseline and PRE to POST changes were measured during treadmill training and overground walking. Results: Individuals with baseline severe walking impairment were at a more significant functional disadvantage across all spatiotemporal walking measures at baseline and demonstrated fewer overall gains post-training. However, contrary to our working hypothesis, both groups experienced comparable increases in cadence, bilateral percent single support times, and step lengths. Conclusion: BLTT is well tolerated and beneficial across all walking impairment levels, and baseline walking speed (≤0.4 m/s) should serve as a covariate in the design of future walking rehabilitation trials.

## 1. Introduction

The consequences of stroke are devastating and often lead to loss of life roles for survivors and their caregivers [1]. This is particularly true for the estimated 20 percent of stroke survivors with severe residual walking impairment [2], who are classified as having a self-selected walking speed lesser or equal to 0.4 m per second [3,4,5]. In contrast to stroke survivors with mild to moderate residual walking impairment (self-selected walking speed >0.4 m/s), individuals with severe walking impairment are more likely to be homebound and are at greater risk of falls [5], fractures, and rehospitalization [6].

While the goal of walking rehabilitation research is to improve the rate and extent of walking recovery across the spectrum of walking severity, current walking rehabilitation interventions do not appear to benefit community ambulators with severe walking impairment to the same extent as ambulators within faster walking subgroups [7,8,9]. For example, the current mainstay of post-stroke walking rehabilitation is treadmill or overground task-specific walking practice [10]. However, the responsiveness to training with this approach has been somewhat limited in community ambulators with chronic severe walking impairment, who often experience little, if any, clinically meaningful benefit. Consequently, a baseline walking speed of ≤0.4 m/s is beginning to gain traction as a predictor of training outcome. Further, some have even advised forgoing walking rehabilitation efforts in this subgroup and instead invest more effort toward teaching severely impaired individuals to utilize devices such as motorized wheelchairs and scooters as primary modes of functional ambulation [7]. While seemingly practical, deemphasizing the use of voluntary mobility and walking rehabilitation training in those with slower walking speed (≤0.4 m/s) may deprive this sizable subgroup of survivors of many inherent benefits of walking practice, such as sustained cognitive and bone health, cardiovascular and respiratory conditioning, and the maintenance of chronic medical comorbidities, all of which are vital to reducing the risk of future vascular events [11,12,13]. As such, there is a need to develop safe, mechanistically sound, effective, and inclusive strategies for stroke walking rehabilitation.

To this end, over the last 15 years, backward walking training (BWT) has been gaining popularity as a potential rehabilitative approach to improve walking performance across disciplines. Studies in athletes [14], the young [15,16], the old [17,18], and individuals with movement disorders [19,20], cerebral palsy [21,22,23,24,25], and stroke [26,27,28,29,30,31,32] have suggested that this training approach can improve various aspects of overground performance such as walking speed, various spatiotemporal measures, and balance. While the mechanistic association between BWT and forward is not well understood, a growing body of work suggests that BWT may be more advantageous than forward walking alone [14,33]. For example, physiologic studies have noted that the backward training approach activates key stability muscles such as the trunk, hip, and knee muscles to a greater extent than forward training [34]. In addition, BWT may facilitate motor control by alleviating the maladaptive flexor-synergy gait pattern typical after brain injury [35,36,37]. From a brain plasticity and functional connectivity perspective, it has been suggested to induce greater cerebral activity in the supplementary motor area, pre-central gyrus, and superior parietal lobule during backward compared with forward walking [38,39].

To further optimize the inherent benefits of the BWT, our group recently tested a protocol termed backward locomotor treadmill training (BLTT). The BLTT protocol differs from past BWT approaches because the entirety of training occurs on a motorized treadmill, without the use of bodyweight support, which may provide greater lower extremity exercise by enabling participants to bear more weight on their paretic limb during training [40]. Furthermore, since participants have to concentrate both on ambulating on a continuously moving belt while simultaneously working to maintain posture and balance in the absence of visual cues, this training protocol may provide greater overall sensory-motor integration training. To this extent, a recent report from our group found that BLTT is safe and feasible across all stroke subgroups, including community ambulators with severe walking impairment [41]. Furthermore, we reported clinically meaningful improvement in walking speed post-intervention and at 2-week follow-up. However, despite the promise of those preliminary findings, critical questions regarding the benefits of BLTT across baseline walking impairment levels (severe (≤0.4 m/s) vs. mild–moderate (>0.4 m/s)) have not been examined. Specifically, it is unknown the degree to which baseline impairment severity may impact training and outcome-related spatiotemporal measures over time. Specifically, this study evaluates how baseline walking impairment level influences training-associated changes in spatiotemporal measures in chronic stroke individuals with severe residual walking impairment compared with those with mild–moderate impairment. Based on previous literature, our working hypothesis was that ambulators with severe post-stroke walking impairment would experience less improvement across all measures relative to participants with mild-moderate impairment. Hence, the objective of this study is to examine the impact walking impairment severity has on training (i.e., BLTT) and subsequent overground walking performance.

## 2. Methods

### 2.1. Design

To address the objectives of this research, a secondary analysis was performed using the dataset from the aforementioned pilot study [41]. For reference, that study aimed to determine the safety, feasibility, and preliminary efficacy of BLTT combined with active versus sham transcutaneous spinal direct current stimulation (tsDCS) in individuals with chronic stroke (*n* = 30) (tsDCS), the specifics which are discussed elsewhere [16,41,42]. We reported that BLTT and tsDCS were safe and tolerable approaches for post-stroke walking rehabilitation training [41]. Surprisingly, contrary to the working hypothesis, there were no significant group differences across all behavioral measures between participants receiving active (*n* = 19) versus sham stimulation (*n* = 11). Following the publication of that report, nine additional subjects (five with severe baseline walking impairment) were recruited directly into the sham group as an internal validation study to rule out the possibility of sampling bias as the cause of the equivocal results (*n* = 19 active tsDCS: 20 sham tsDCS); nevertheless, the results remained unchanged. Therefore, since there were no substantial differences between groups ((BLTT + sham/anodal tsDCS) (see Supplementary Table S1)), the dataset was combined to address the objectives of this study.

### 2.2. Setting and Participants

This study was approved by the University of Cincinnati Institutional Review and was performed in the Neurorecovery Lab from September 2017 to October 2019. Study participants were greater than 6 months post-stroke (chronic) and recruited from the community. As previously described [41], all participants provided written informed consent prior to enrollment, according to the Declaration of Helsinki recommendations. Inclusion and exclusion criteria were previously described elsewhere. They included: 18–80 years of age, residual walking impairment secondary to ischemic/hemorrhagic stroke(s), ambulating at least 10 m without a walker, and maintaining at least a 0.13 m/s speed on the treadmill while walking in a backward direction for six consecutive minutes. In addition, all participants were asked to abstain from formal physiotherapy through the entirety of training and follow-up. No botulinum toxin injections were permitted at least 2 weeks prior to study enrollment and the follow-up visit. Exclusion criteria: unstable cardiovascular status precluding participation in moderate–high intensity exercise, severe lower extremity spasticity (modified Ashworth >2/4), significant language barrier that may interfere with the ability to follow instructions during training and testing, and untreated depression [>10 on the Patient Health Questionnaire (PHQ9)] [43]. In total, 28 individuals were classified as having mild–moderate and 11 with severe walking impairment based on baseline preferred 10-mWT speed obtained at the screening visit. There were no significant differences in baseline demographics (gender, age, height, cognition). Of note, there was a significant difference in stroke chronicity due to six individuals in the mild–moderate group who were greater than 100 months from stroke onset, compared with zero in the severe group (Table 1).

### 2.3. Description of Training and Outcomes

#### Backward Locomotor Treadmill Training (BLTT)

**Figure 1:**
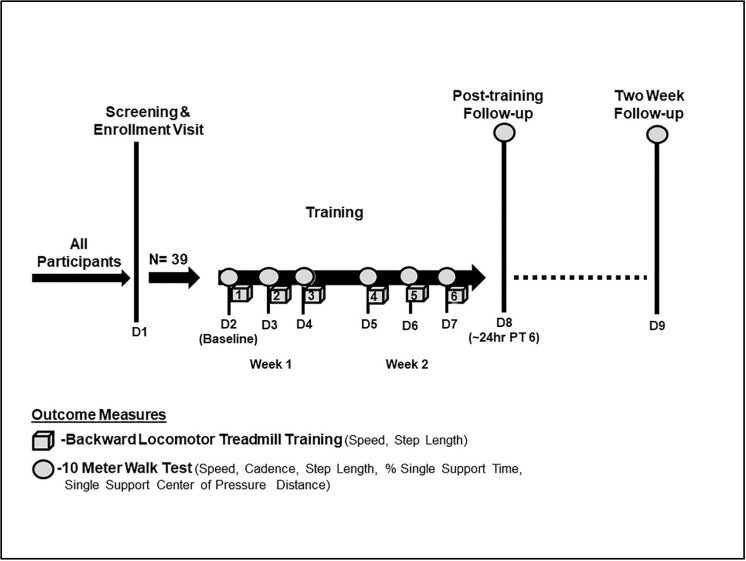
Backward locomotor treadmill training (BLTT) protocol and outcome measures. Study participants underwent six 30-min sessions of BLTT over 2 weeks (cube). The BLTT-related outcome measures were obtained during the six sessions of training (D2–D7). In contrast, outcome measures related to overground walking performance were obtained prior to each training session (baseline (D2), subsequent training days (D3–D7)), ~24 h following the completion of the sixth training day (D8), and at 2-week follow-up.

On the first visit (screening), enrolled participants were oriented to the backward locomotor treadmill task while holding one handrail for support for 3 min. Regardless of the level of walking impairment, all participants were expected to maintain the minimum required training speed of 0.13 m/s on the instrumented Biodex Gait Trainer™ 3 motorized treadmill, as this speed was the minimum necessary for the treadmill sensors to detect and record walking metrics associated with training. As previously described, the belt speed was increased in increments of +0.04 m/s until a comfortable training speed was achieved [41]. Qualifying participants then underwent six sessions of training, which consisted of four 6-min blocks. All training sessions were conducted by a protocol-trained and certified physical therapist (Figure 1). The use of baseline orthotics was allowed during training. The starting belt speed was based on the last preferred speed achieved on the previous day, with the option to increase or reduce the speed per subject preference. For additional safety, all participants wore a safety harness (without body weight support) and were provided 2-min rest breaks between each 6-min training block. Participants were cued to step “reach” back as far as possible with each step during the swing phase of gait while working to maintain an upright posture throughout the duration of the training period. In addition, to reduce possible confounders between training sessions, participants were instructed not to practice walking backward outside of the study protocol for the duration of the study.

### 2.4. Outcomes

#### 2.4.1. BLTT

Training-related outcome variables were baseline and PRE to POST changes in backward walking speed and step lengths (paretic and nonparetic). The use of a baseline assistive device and orthotics were allowed during the assessment. Walking speed is the average speed of the treadmill belt during each training session. An increase in this measure may suggest improved confidence, stability, and greater neuromotor control [44]. Likewise, the change in step length has been postulated to be associated with lower extremity strengthening and motor control and often declines with age and central nervous system injury [18,45]. Hence, investigating training-related changes in these measures could inform how training strategies differ between walking impairment levels. The backward walking speed and step lengths were acquired using built-in treadmill sensors [46] and were later exported for offline analysis. Four separate values (from each 6-min training block) were averaged to formulate a single cumulative value per training session. Of specific interest were group differences in baseline (D2) performance and PRE to POST changes (D7–D2). Information regarding study adverse events were documented throughout the study. In addition, a tolerability and safety questionnaire was completed by the patient at the first post-training follow-up (D8) and asked participants to rate (0 = None to 10 = Severe) their level of soreness and fatigue related to BLTT.

#### 2.4.2. Overground Walking (10-m Walk Test—Fast)

Participants were instructed to walk as fast as possible, with or without an assistive device (single-point cane or quad cane), with the fastest of three attempts used for analysis. Five measures were obtained during the 10-mWT-fast: speed, cadence, and bilateral (paretic and nonparetic) step lengths, percent single support times (%-SST), and single support center of pressure distances (SS COP Dist.). Forward walking speed is a valid, reliable, and good predictor of functional ambulation and community independence [3] and is commonly impaired after stroke. Cadence is defined as the number of completed steps per minute, negatively impacted with aging after stroke [47], and may inform of training changes in motor control [45,48]. Likewise, bilateral step length significantly decreases with age and after stroke and is associated with decreased walking speed and an increased risk of falls [49,50]. The %-SST is a well-validated temporal measure of single stance and is associated with changes in lower extremity strength, stability, step length, and walking speed [51,52]. Lastly, the SS COP Dist. is a spatial measure of how bodyweight progresses over the foot during single support and has been suggested to be a predictor of walking ankle–foot stability and speed [53,54]. As such, measurement of training-related changes in SS COP Dist. may provide information about the neuromuscular response involved in maintaining upright balance and forward progression during walking. To obtain these measures, the 10-mWT—*Fast* was performed daily prior to the start of BLTT and was captured with a 20-feet Zeno Walkway gait analysis mat (Protokinetics, PA, USA) and Protokinetics Movement Analysis Software (PKMAS) [25], centered 2 m from the starting point of the 10-mWT and were later exported for offline analysis. Of specific interest were: between-group differences at baseline (D2), PRE to POST changes (D8-D2), and retention at 2 weeks follow-up (D9-D8).

### 2.5. Statistical Analysis

A total of 39 participants were included in the analysis for BLTT. For overground walking performance on the 10-mWT, one participant was excluded from overground walking analysis for run-walking, which made the data uninterpretable. Shapiro–Wilk tests were used to assess for deviations from normal distribution among the variables, and the significance level was set at *p* = 0.05 for all measures. Linear mixed-effects models for each outcome measure accounting for repeated measures on the same individual with a categorical visit, baseline severity indicator (mild–moderate versus severe), and visit-by-severity interaction terms was used to test baseline differences, PRE to POST changes, and retention of spatiotemporal measures (forward overground walking (D9-D8)). All analyses were conducted using SAS version 9.4 (Cary, NC, USA).

## 3. Results

### 3.1. Safety and Tolerability

There were no adverse events throughout the 4-week duration of the study. With reference to BLTT-related tolerability and discomfort (0 = None, 10 = Severe), both groups reported zero to minimal training-related soreness, with the mild–moderate group reporting 1.41 ± 1.85, median-0, compared with the severe walking group (1.45 ± 2.16, median-0), *p* = 0.946. In addition, both groups experienced zero to minimal training-related fatigue; however, the mild–moderate group reported less fatigue (1.33 ± 1.84, median-0) compared with the severe group (3.45 ± 3.01, median-3), *p* < 0.012.

### 3.2. BLTT—Speed

**Figure 2:**
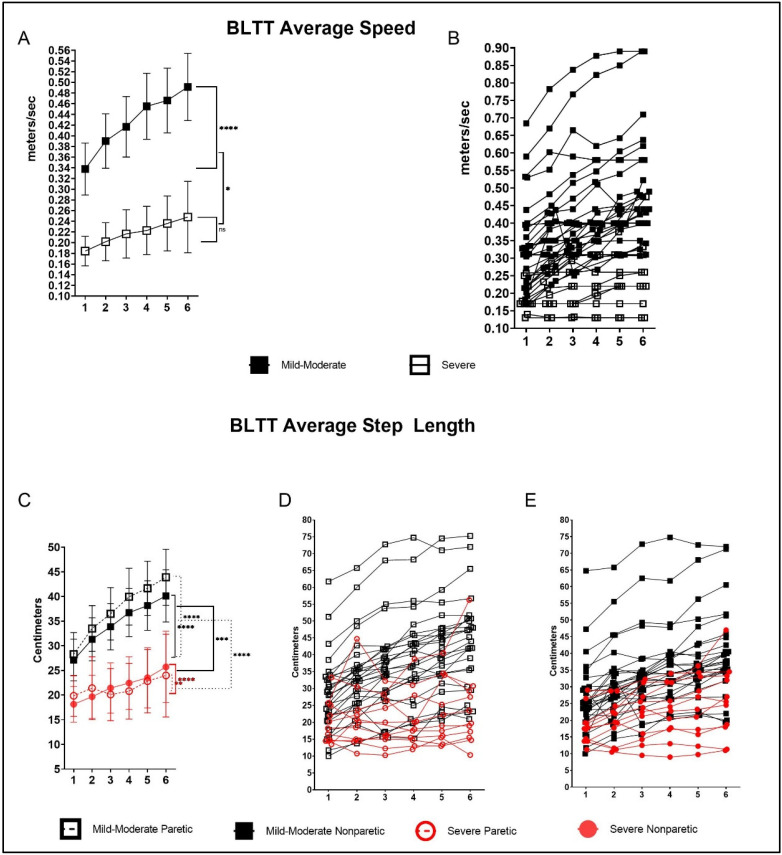
Backward locomotor training (BLTT) speed and step length over six training sessions. Mean progression in BLTT speed per training session from baseline session 1 (D2) through 6 (D7 (**A**); and individual values (**B**) paretic and nonparetic step lengths (**C**); and individual values; paretic (**D**), and nonparetic step lengths (**E**). Error bars show 95% CI; * *p* < 0.05, ** *p* < 0.01, *** *p* < 0.001, **** *p* < 0.0001.

Baseline training speed (mean (95% CI)) for the mild–moderate group was faster (0.34 m/s (0.30, 0.37)) compared with the severe group (0.18 m/s (0.13, 0.24)), *p* < 0.001. Both groups demonstrated improvement in PRE to POST change in BLTT walking speed, with the mild-moderate group showing greater improvement (Δ 0.15 m/s (0.11, 0.19; *p* < 0.001)) relative to the severe group, which improved but did not reach a level of significance (Δ 0.06 m/s (−0.00, 0.13); *p* = 0.055). There was a significant between-group difference in magnitude of change in PRE to POST BLTT walking speed (*p* = 0.027; Figure 2A).

### 3.3. BLTT—Paretic Step Length

Baseline paretic step length was longer in the mild–moderate group (28.3 cm (23.9, 32.7)) compared with the severe group (19.8 cm (12.9, 26.8)). Both groups demonstrated improvement in PRE to POST paretic step length, with the magnitude of change being greater in the mild–moderate group (Δ 15.2 cm (13.5, 16.9); *p* < 0.001) relative to the severe group (Δ 4.14 cm (1.55, 6.73); *p* < 0.002)), *p* < 0.001 (Figure 2B).

### 3.4. BLTT—Nonparetic Step Length

Baseline nonparetic step length was longer in the mild–moderate group (27.1 cm (23.1, 31.1)) compared with the severe group (18.1 cm (11.8, 24.4)). Both groups demonstrated improvement in PRE to POST paretic step length, with the magnitude of change being greater in the mild–moderate group (Δ 12.5 cm (11.05, 14.1); *p* < 0.001) relative to severe group (Δ 7.58 cm (5.16, 9.99); *p* < 0.001)), *p* < 0.001 (Figure 2B).

### 3.5. Overground Walking Performance

#### 3.5.1. 10-mWT Speed (Fast)

**Figure 3:**
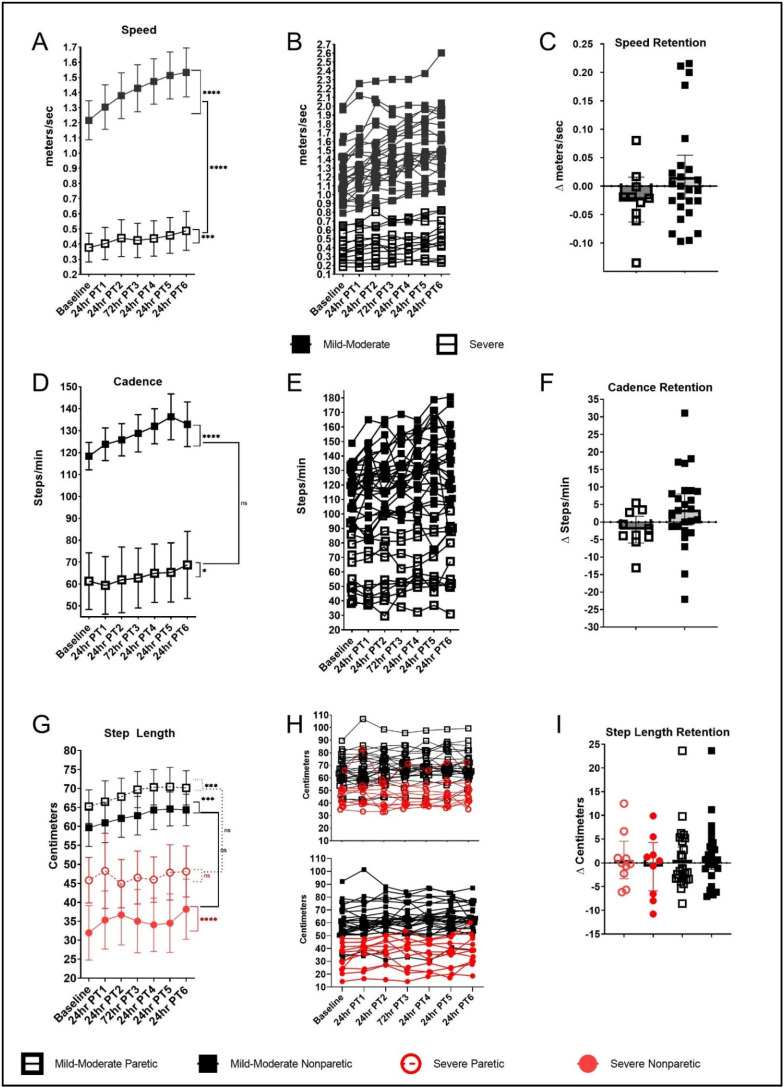
Overground walking performance (speed, cadence, step length). Mean progression in 10-mWT (*fast*) speed (**A**), individual values (**B**) and retention (**C**); cadence (**D**), individual values (**E**), and retention (**F**); paretic and nonparetic step lengths (**G**), individual values (**H**), and retention (**I**). Error bars show 95% CI; * *p* < 0.05; *** *p* < 0.001; **** *p* < 0.0001.

Baseline 10-mWT speed was faster in the mild–moderate group (1.22 m/s (1.09, 1.34)) compared with the severe group (0.38 m/s (0.18, 0.58)), *p* < 0.001. Both groups demonstrated improvement in PRE to POST changes in forward overground walking speed, with the magnitude of change being greater in the mild–moderate group (Δ 0.29 m/s (0.25, 0.34); *p* < 0.001)) relative to the severe group (Δ 0.11 m/s (0.04, 0.18); *p* < 0.002), *p* < 0.001 (Figure 3A). Both groups demonstrated retention of gains in walking speed at the 2-week follow-up (mild–moderate, *p* = 0.489; severe, *p* = 0.391; Figure 3B).

#### 3.5.2. 10-mWT—Cadence

Baseline cadence for the mild and moderate group was higher (118.4 (110.4, 126.4)) compared with the severe group (61.32 (48.79, 73.86)), *p* < 0.001. Both groups demonstrated significant improvement in PRE to POST changes in cadence (mild–moderate group, Δ 14.74 steps/min (10.16, 19.32), *p* < 0.001; severe group, Δ 7.417 steps/min (0.424, 14.41), *p* = 0.038), with no between-group differences in the magnitude of change, *p* = 0.089 (Figure 3C). Both groups demonstrated retention of gains in cadence at the 2-week follow-up (mild–moderate, *p* = 0.130; severe, *p* = 0.381; Figure 3D).

#### 3.5.3. 10-mWT—Paretic Step Length

Baseline 10-mWT paretic step length for the mild–moderate group was longer (65.2 cm (60.6, 69.8)) compared with the severe group (45.8 cm (38.6, 53.0)), *p* < 0.001. The mild–moderate group experienced an improvement in PRE to POST change in forward paretic step length (Δ 3.54 cm (1.27, 5.81); *p* = 0.002) and demonstrated retention at the 2-week follow-up (*p* = 0.576). There was no significant PRE to POST change in forward paretic step length in the severe group (Δ 2.30 cm (−1.17, 5.77); *p* < 0.193). However, there were no significant differences in the magnitude of change in PRE to POST changes in paretic step length (*p* = 0.556) between the mild–moderate and severe groups (Figure 3E,F).

#### 3.5.4. 10-mWT—Nonparetic Step Length

Baseline 10-mWT nonparetic step length for the mild–moderate group was longer (59.6 cm (55.0, 64.3)) compared with the severe group (31.9 cm (24.7, 39.2)), *p* < 0.001. Both groups demonstrated significant improvement in PRE to POST changes in nonparetic step length (mild–moderate group, Δ 3.58 cm (1.28, 5.88), *p* = 0.002; severe group, Δ 6.23 cm (2.73, 9.74), *p* < 0.001), with no between-group differences in the magnitude of change, *p* = 0.21. Both groups demonstrated retention in nonparetic step length gains at the 2-week follow-up (mild–moderate, *p* = 0.239; severe, *p* = 0.390).

#### 3.5.5. 10-mWT—% SST Paretic

**Figure 4:**
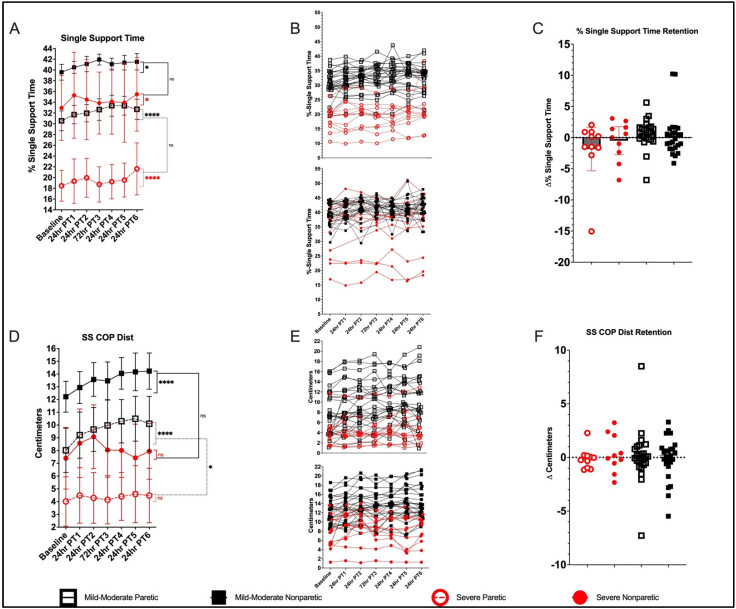
Overground walking performance (SST, SS COP Dist.). Mean progression in 10-mWT (*fast*) %SST paretic and nonparetic (**A**) individual values (**B**) and retention (**C**); SS COP Dist paretic and nonparetic (**D**), individual values (**E)** and retention (**F**). Error bars show 95% CI; * *p* < 0.05; **** *p* < 0.0001.

Baseline 10-mWT % SST paretic was greater in the mild–moderate group (30.6% (28.8, 32.3)) compared with the severe (18.5% (15.7, 21.3)), *p* < 0.001. Both groups demonstrated significant improvement in PRE to POST changes in paretic % SST (mild–moderate group, Δ 1.99% (1.00, 2.97), *p* < 0.001; severe group, Δ 3.13% (1.63, 4.64), *p* < 0.001), with no between-group differences in the magnitude of change, *p* = 0.21 (Figure 4A). The mild–moderate group demonstrated retention at the 2-week follow-up (*p* = 0.218), but a regression of gains was observed in the severe group (*p* = 0.020; Figure 4B).

#### 3.5.6. 10-mWT—% SST Nonparetic

Baseline 10-mWT% SST nonparetic was greater in the mild–moderate group (39.6% (37.3, 41.9)) compared with the severe group (32.9% (29.4, 36.4)), *p* < 0.001. Both groups demonstrated significant improvement in PRE to POST changes in nonparetic % SST (mild–moderate group, Δ 1.76% (0.42, 3.09), *p* = 0.01); severe group, Δ 2.54% (0.51, 4.58), *p* = 0.01), with no between-group differences in the magnitude of change, *p* = 0.52. Both groups demonstrated retention of %-SST nonparetic gains at the 2-week follow-up (mild–moderate, *p* = 0.659; severe, *p* = 0.308).

#### 3.5.7. 10-mWT—SSCOP Dist. Paretic

Baseline SSCOP Dist. paretic was longer in the mild–moderate group (8.02 cm (6.43, 9.62)) compared with the severe group (4.01 cm (1.52, 6.51)), *p* < 0.001. The mild–moderate group experienced an improvement in PRE to POST changes in SSCOP Dist. paretic (Δ 1.97 cm (1.23, 2.71), *p* < 0.001) and demonstrated retention at the 2-week follow-up (*p* = 0.746). No significant PRE to POST change was observed in the severe group (Δ 0.47 cm (−0.66, 1.59), *p* = 0.42). There was a significant between-group difference in magnitude of PRE to POST change in paretic SSCOP Dist. (*p* = 0.029).

#### 3.5.8. 10-mWT—SSCOP Dist. Nonparetic

Baseline SSCOP Dist. nonparetic was longer in the mild–moderate group (12.2 cm (10.9, 13.6)) compared with the severe group (7.40 cm (5.27, 9.52)), *p* < 0.001. The mild–moderate group experienced improvement in PRE to POST change in SSCOP Dist. nonparetic (Δ 1.74 cm (1.04, 2.44), *p* < 0.001) and demonstrated retention at the 2-week follow-up (*p* = 0.780). No significant PRE to POST change was observed in the severe group (Δ 0.55 cm (−0.52, 1.61), *p* = 0.31). There was no significant between-group difference in magnitude of PRE to POST change in paretic SSCOP Dist. (*p* = 0.067).

## 4. Discussion

Our results indicate that stroke survivors with severe baseline walking impairment are at a significant functional disadvantage across all tested spatiotemporal walking measures than those with mild–moderate impairment. Nevertheless, in contrast to the working hypothesis, our findings suggest that the group with severe baseline walking impairment tolerated and still benefited from BLTT based on their training-related improvements on several measures, including BLTT and overground forward walking speeds, cadence, step lengths, and percent single support times. Of particular significance were the improvements in cadence, single support time of the paretic leg, and nonparetic step length, which were comparable in magnitude to changes observed in the mild–moderate group. Furthermore, those gains were retained up to the 2-week follow-up period, reducing the likelihood that these findings resulted from repeated testing (i.e., practice).

From a rehabilitation training standpoint, the BLTT protocol appears beneficial across groups. Past studies have hypothesized that backward walking training, particularly when performed on the treadmill, facilitates lower extremity strengthening [55] and enhances proprioception [56,57], agility, and balance [17] while enabling aerobic conditioning [58]. Furthermore, since participants cannot see where their feet are in space while walking on a moving treadmill platform, such a task can be both physically [45] and cognitively [38,59] demanding. Therefore, it is likely that participants may benefit from the repetitive practice of this approach in a safe and monitored setting.

While the improvements observed in the severe group were not at the magnitude noted in the mild-moderate cohort for clinical measures like overground walking speed, the changes observed after just six training sessions are reassuring and warrant further investigation to determine how outcomes can be augmented. For example, the mean PRE to POST change in the 10-mWT (fast) was 0.11 m/s, surpassing levels achieved in several rigorous walking rehabilitation studies (with longer training times) [60], and approaching the benchmark for the minimal clinically important difference (MCID = 0.16 m/s) for post-stroke walking recovery [61]. Furthermore, improvements were not limited to walking speed but also included gains in several spatiotemporal measures such as cadence, bilateral %-SSTs, and non-paretic limb step lengths. While the factors influencing these changes are likely multifactorial, we postulate that successive BLTT resulted in an improved increase in power output and stability in both groups.

In contrast to the above, this study found that only the mild-moderate group experienced an improvement, albeit minimal, in bilateral SSCOP Dist., while no significant changes were recorded in the severe group. This suggests that foot-ankle stability, determined by SSCOP Dist., remained clinically impaired in the severe group following six training sessions. Nevertheless, based on the observed upward trend observed after six training sessions, it would be instructive to determine if additional training sessions may improve this measure and further improve walking performance. To this end, previous studies have suggested that individuals with severe walking deficits may require a more extended period of training and time to show even greater meaningful benefit from intervention [5,62], as may be the case for BLTT. Another possibility is that some outcome changes may be proportional to baseline function. For example, percent changes were similar between subgroups in some cases where absolute changes differed.

### Limitations

Since this study was a secondary analysis of a safety and feasibility pilot study, our findings are not definitive and did not adjust for multiple comparisons. Therefore, future well-powered studies are needed to validate the magnitude of improvement in walking speed and other spatiotemporal measures observed in this study. Moreover, the study did not include BWT or forward walking controls; therefore, it was not possible to distinguish what changes, if any, are specific to BLTT compared with other interventions. Hence, future studies are needed to empirically test differences between BLTT and other aerobic and strength training interventions. In addition, this study was limited to ambulators; therefore, our findings are not generalizable to non-ambulatory stroke survivors, for whom BLTT is likely not feasible without bodyweight support. Furthermore, our findings are limited to what was observable within six training sessions, and it is possible that some of the changes in the biomechanical properties of walking, such as SSCOP Dist., may take longer to develop. Additionally, the severe group was significantly smaller, and it is possible that the inclusion of additional participants might have influenced the outcome. Although beyond the scope of this paper, past studies have suggested that changes in walking speed, cadence, and step/stride length are codependent, suggesting that an increase in one inherently increases the other [63]. Hence, while this study highlights training-related changes, it is limited in its ability to identify the underlying mechanisms for these changes (e.g., increased foot propulsive force, cerebral, or musculoskeletal activation). Therefore, future studies that incorporate dynamometric measures and dynamic peripheral and cerebral electrophysiologic markers are needed.

## 5. Conclusions

Our findings suggest that BLTT is well tolerated and results in progressive improvement in walking speed and other spatiotemporal measures during training and overground walking but to a lesser extent in individuals with severe walking impairment on some measures. However, both groups experienced comparable increases in cadence, bilateral percent single support times, and step lengths. Therefore, future backward walking training studies should include individuals with severe walking impairment and incorporate baseline comfortable walking speed (≤0.4 m/s) as a covariate in their design.

## Figures and Tables

**Table 1:** Baseline demographics.

	Groups (Severity)	Demographics							
		Gender	Height(cm)	Age (Yr)	MMSE	Stroke Location	Type (I vs. H)	Stroke Age (Mo)	Assistive Device
**Mild–Moderate (>0.4 m/s)**	BLTT-3	F	162	47.43	29	L MCA	I	37.80	AB
	BLTT-7	F	170	51.12	27	R MCA	I	76.71	AFO
	BLTT-9	M	182	56.04	29	L Basal Ganglia	I	172.5 *	
	BLTT-11	M	172	69.81	28	L PCA	I	36.53	-
	BLTT-14	M	182	65.11	28	R Basal Ganglia	I	33.18	-
	BLTT-19	M	177	64.51	25	L ACA/MCA	I	140.3 *	-
	BLTT-21	F	172	54.79	30	L MCA	I	12.42	AFO
	BLTT-48	M	182	57.85	27	LMCA	I	11.31	-
	BLTT-2	F	167	38.01	28	L MCA/PCA	H	68.13	AFO
	BLTT-4	M	182	62.32	29	R ACA	I	147.6 *	-
	BLTT-5	M	180	33.68	30	L > R Midbrain	H	11.71	KFO, KB
	BLTT-8	M	180	63.34	26	R MCA	I	100.0 *	AFO
	BLTT-12	F	157	63.46	26	L MCA	I	222.6 *	-
	BLTT-13	M	175	56.93	29	R MCA	I	18.42	-
	BLTT-17	F	172	44.07	28	R MCA	I	8.433	-
	BLTT-20	F	170	49.69	30	R MCA	H	41.45	SKC, AFO
	BLTT-23	M	167	66.78	30	L Cerebellar	I	9.806	-
	BLTT-24	F	162	51.52	29	L MCA	I	9.258	AFO
	BLTT-26	M	193	57.27	28	L Basal Ganglia	I	18.23	-
	BLTT-29	M	195	72.92	28	R > L Cerebellar	I	18.94	-
	BLTT-30	M	177	54.73	29	L MCA	H	48.10	-
	BLTT-32	F	160	63.02	27	L MCA	I	166.1 *	-
	BLTT-34	F	175	64.93	30	R Pontine	I	29.23	-
	BLTT-36	F	160	69.35	28	L Pontine	I	10.13	-
	BLTT-39	F	162	62.16	29	R MCA	I	82.23	-
	BLTT-40	F	170	34.73	29	R MCA	I	28.61	-
	BLTT-44	F	162	61.33	30	R MCA	I	74.24	C
	BLTT-46	M	170	69.60	29	R Basal Ganglia	I	26.01	-
	Mean ± SD		172.7 ± 9.684	57.38 ± 10.52	28.39 ± 1.343			59.28 ± 59.25	
**Severe (≤0.4 m/s)**	BLTT-1	F	167	58.11	25	L MCA	I	49.40	WBQC
	BLTT-16	M	172	56.74	30	L Basal Ganglia	I	12.39	AFO
	BLTT-25	M	180	58.07	28	L MCA	I	9.226	C, AFO
	BLTT-28	M	193	53.31	30	R MCA	H	25.94	C, AFO
	BLTT-33	F	165	55.93	30	R MCA	I	20.90	NBQC
	BLTT-37	F	167	52.98	29	R ACA/Pontine	H	11.03	HC, AFO
	BLTT-38	M	177	47.70	21	L MCA	H	8.548	HW, AFO
	BLTT-41	M	177	55.51	28	R MCA	I	34.13	NBQC, AFO
	BLTT-42	M	175	64.47	30	R MCA	H	83.31	WBQC
	BLTT-47	M	187	38.69	29	R MCA	I	11.28	NBQC, KFO
	BLTT-49	M	180	69.73	29	R MCA	I	28.83	WBQC, AFO
	Mean ± SD		176.4 ± 8.617	55.57 ± 8.078	28.09 ± 2.773			26.82 ± 22.66	
	*p*-value		0.132	0.475	0.737			0.009	

* Indicates greater or equal to 100 months from stroke event; MMSE, Mini-Mental State Exam; I, ischemic; H, hemorrhagic; AB, ankle brace; AFO, ankle foot orthotic; C, cane; HC, hemi-cane; KB, knee brace; KFO, knee foot; NBQC, narrow-based quad cane; SKC, Swedish knee cage; WBQC, wide-based quad cane.

## Data Availability

The data presented in this study are available on request from the corresponding author.

## Supplementary Materials

The following are available online at https://www.mdpi.com/article/10.3390/brainsci12020133/s1, Table S1: Between Group Comparison of Outcomes.
