# Supplementary material for: Backward Locomotor Treadmill Training Differentially Improves Walking Performance across Stroke Walking Impairment Levels
Source: Brain Sci. 2022 Jan 19;12(2):133. doi: 10.3390/brainsci12020133 (PMC8870096; doi:10.3390/brainsci12020133)
Supplement: Supplementary file 1 [file brainsci-12-00133-s001.zip › brainsci-1511988-supplementary.pdf]

Supplementary Table S1. Between Group Comparison of Outcomes

Outcome Measures	BLTT+ Anodal tsDCS (N=19)	BLTT+ Sham tsDCS (N=20)	P-Value
	Δ POST-PRE (*mean ± SEM)*		
**BLTT**			
Speed (m/s)	0.127 ± *0.012*	0.122 ±*0.012*	0.773
Step Length $_{Paretic}$ *(cm)*	11.35 ± *1.388*	12.09 ±*1.282*	0.698
Step Length $_{Nonparetic}$ *(cm)*	10.99 ±*1.137*	11.27±*1.050*	0.855
**10-Meter Walk Test**			
Speed (m/s)	0.218 ± *0.028*	0.253 ± *0.027*	0.382
Cadence (steps/min)	13.08 ±*3.815*	15.66 ±*4.410*	0.662
Step Length $_{Paretic}$ *(cm)*	3.658 ± *1.405*	2.870± *1.330*	0.684
Step Length $_{Nonparetic}$ *(cm)*	3.123 ± *1.393*	5.320 ± *1.319*	0.253
SS COP Dist $_{Paretic}$ *(cm)*	1.609 ± *0.526*	1.543± *0.621*	0.937
SS COP Dist $_{Nonparetic}$ *(cm)*	0.741± *0.448*	2.014± *0.522*	0.076

BLTT-Backward Locomotor Treadmill Training; SS COP Dist.- Single Support Center of Pressure Distance; % SST- Percent Single Support Time
